# How Exploitative Leadership Emerges: The Activating Effect of Organizational Context on Individual Traits

**DOI:** 10.3390/bs15081093

**Published:** 2025-08-12

**Authors:** Junhui Zhang, Yilin Zhang, Wei Pan

**Affiliations:** 1 School of Management, Guangdong Ocean University, Zhanjiang 524088, China; zhangjh0816@gdou.edu.cn; 2 School of Economics and Management, Harbin Engineering University, Harbin 150001, China; panwei@hrbeu.edu.cn

**Keywords:** exploitative leadership, formation mechanism, trait activation, personality traits, organizational contexts

## Abstract

Although the detrimental effects of exploitative leadership have been widely revealed, scholarly understanding of its formation mechanisms remains insufficient. Addressing this gap, the present study draws upon trait activation theory to investigate the antecedents and formation mechanisms of exploitative leadership through the dual lenses of personality traits and organizational contexts. Through an empirical examination of questionnaire data from 422 leader–subordinate dyads in Chinese organizations, the results of this study reveal that: three leader personality traits—self-interest, other-oriented perfectionism, and high power distance orientation—significantly and positively predict exploitative leadership behaviors; three organizational contexts—task challenge, flexible status conferral, and leader–subordinate goal misalignment—likewise serve as positive predictors of exploitative leadership behaviors; through trait activation effects, flexible status conferral significantly strengthens the positive relationships between all three personality traits and exploitative leadership, while task challenge and goal misalignment specifically amplify the positive relationships of exploitative leadership with high power distance orientation and self-interested personality, respectively. The research findings are of great value for improving the theoretical framework of exploitative leadership and guiding management practice.

## 1. Introduction

The phenomenon of leaders leveraging their positions to oppress and exploit subordinates for personal gain is not uncommon in today’s workplace, posing significant harm. In academia, such leadership behavior is termed “exploitative leadership” (Schmid et al., 2019). As a typical form of negative leadership, exploitative leadership has been proven to have significant effects on both organizations and employees (Ye et al., 2022; Z. N. Wang et al., 2024b; Z. N. Wang et al., 2023b). Against this backdrop, uncovering the causes of exploitative leadership and curbing its emergence at the source is of paramount importance.

However, although scholars have identified some antecedents of exploitative leadership, such as leaders’ dark personalities (Cohen & Baruch, 2022; Lazreg & Lakhal, 2022), workplace ambition (Z. N. Wang et al., 2024a), performance pressure (Deng et al., 2023), high perceptions of goal blockages (Emmerling et al., 2023), and perceptions of power and seniority (L. M. Guo et al., 2024; Z. N. Wang et al., 2024a), these findings remain insufficient. Some key influencing factors, such as leaders’ individual high-power distance orientation and perfectionist personality, as well as organizational contextual factors like leader–subordinate goal misalignment and the flexible conferral of status within organizations, have yet to be explored or verified as drivers of exploitative leadership, along with their underlying mechanisms.

**Figure 1:**
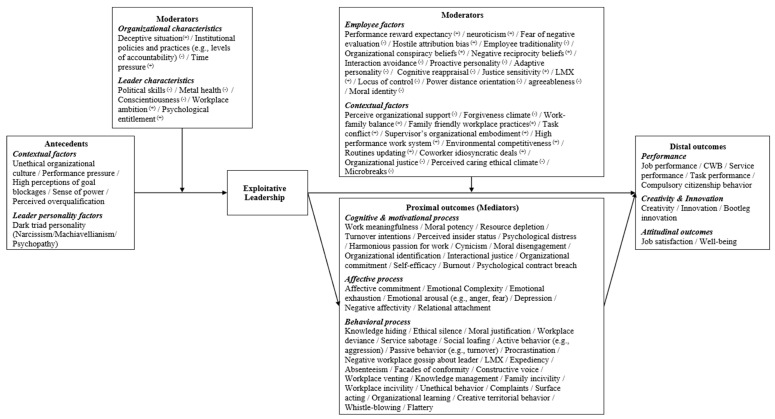
Summary of research results on exploitative leadership. Note: This figure was compiled by the authors’ teams (Abdulmuhsin et al., 2021; Akhtar et al., 2022; Akram et al., 2024; A. Bajaba et al., 2023; S. Bajaba et al., 2022; Basaad et al., 2023; Cheng et al., 2023; Cohen & Baruch, 2022; Deng et al., 2023; Deng & Wen, 2023; Elsaied, 2022; Emmerling et al., 2023; Fatima & Majeed, 2023; Feng et al., 2022; Gao et al., 2022; Garlatti Costa et al., 2022; L. M. Guo et al., 2021; L. M. Guo et al., 2024; L. Y. Guo et al., 2024; Huang & Tang, 2020; Huang & Tang, 2024; Khursheed, 2020; Kiyani et al., 2021; Lazreg & Lakhal, 2022; M. W. Liu et al., 2023; Luo et al., 2021; Lyu et al., 2023; Majeed et al., 2023; Moin et al., 2024; Schmid et al., 2018; Schmid et al., 2019; X. Sun et al., 2024; Y. D. Sun & Zhang, 2022; Z. Z. Sun et al., 2023; Syed et al., 2021; Y. L. Wang et al., 2024; Z. N. Wang et al., 2024a; Z. N. Wang et al., 2023a; Z. N. Wang et al., 2024b; Z. N. Wang et al., 2021a; Z. N. Wang et al., 2021b; Z. N. Wang et al., 2024c; Wei et al., 2023; Wu et al., 2021; Ye et al., 2023; Ye et al., 2022; Zahur et al., 2022; Y. J. Zhang et al., 2022; Y. J. Zhang et al., 2023).

Furthermore, as shown in Figure 1, compared to the abundant research achievements on the consequences of exploitative leadership, the academic exploration of its causes and formation mechanisms still leaves considerable room for improvement. In light of this, the current paper investigates the causes and formation mechanisms of exploitative leadership, aiming to advance the theoretical research on exploitative leadership toward greater completeness.

Regarding the antecedents of individual behavior, the interactionist perspective in organizational behavior suggests that individual behavior is typically the result of the combined influence of personal factors and situational factors, with personality traits often regarded as the most stable and effective drivers of individual behavior. Building on this, trait activation theory posits that whether personality traits are expressed in an individual’s work behavior depends on whether the situational cues faced by the individual effectively “activate” those traits (Tett & Burnett, 2003). When activated by situational cues, personality traits are more likely to manifest in work behavior, resulting in a stronger correlation between the two. Conversely, when suppressed by situational cues, the correlation between personality traits and specific work behaviors tends to be weaker. Based on the above theories, this study explores the antecedents of exploitative leadership from two dimensions: leader personality traits and organizational context, and further investigates the activating effect of organizational context on leader personality traits to better identify the formation mechanism of exploitative leadership.

Regarding leader personality traits, this study examines the predictive effects of leaders’ self-interested personality, other-oriented perfectionism, and high-power distance orientation on exploitative leadership behavior, as these factors encompass the core motivations, relationships and control methods, as well as the cognitive legitimacy basis of exploitative leadership, collectively forming the “susceptible combination” that makes leaders prone to engaging in exploitative behavior. In terms of contextual factors, the study selects task challenge, leader–subordinate goal misalignment and flexible status conferral as situational cues at the task level, social level, and organizational level, respectively, in order to explore their predictive effects on exploitative leadership and their moderating roles in activating leader’s personality traits and strengthening the relationship between these traits and exploitative leadership behavior.

Through empirical examination of questionnaire data from 422 leader–subordinate dyads in Chinese organizations, the current paper provides a great answer to the question of “how exploitative leadership emerges”. The research findings not only help fill the current academic gap regarding the antecedents of exploitative leadership and contribute to the further development of theoretical research in this domain, but also offer practical guidance for organizations. Specifically, they assist enterprises in formulating targeted intervention strategies to effectively curb the emergence of exploitative leadership, thereby optimizing leadership practices within corporate settings.

The paper is structured into several sections. In the theoretical basis and research hypotheses section, we analyze the predictive effects of three personality traits and three contextual factors on exploitative leadership based on trait activation theory, examine the trait activation effects involved, propose research hypotheses, and construct a theoretical model. In the materials and methods section, we specify the sources of empirical data, data collection procedures, and measurement instruments used in this study. In the results section, we present the empirical testing outcomes of our research hypotheses. In the discussion section, we summarize the research findings, elaborate on the theoretical contributions and practical implications of this study, while also identifying limitations of current research and suggesting future directions. In the conclusion section, we provide a final summary of this research.

## 2. Theoretical Basis and Research Hypotheses

### 2.1. The Predictive Effect of Leader Personality Traits on Exploitative Leadership Behavior

Personality traits serve as crucial factors in explaining individual behavior. The following section examines how leaders’ personality traits—namely, self-interested personality, other-oriented perfectionism, and high-power distance orientation—predict exploitative leadership behavior.

A self-interested personality refers to a self-centered trait characterized by excessive focus on personal gain while neglecting or disregarding the feelings and needs of others (Weigel et al., 1999). Its typical manifestations include selfishness, greed, stinginess, sensitivity, and distrust. Individuals with a self-interested personality tend to dismiss others, exploit them, and shift blame onto them, making them more likely to engage in unethical workplace behaviors. When leaders possess a self-interested personality, they often prioritize their own interests over those of the team or organization, leveraging their power and status to manipulate subordinates. They may even resort to deception, threats, or intimidation to compel compliance. When facing difficulties or failures, such leaders tend to blame their subordinates, whereas when the team or organization achieves success, they exaggerate their own contributions while overlooking the efforts of team members. In summary, a leader’s self-interested personality drives them to engage in exploitative behavior toward subordinates. Accordingly, we propose the following hypothesis:

**H1:**  *A leaders’ self-interested personality positively predicts their exploitative leadership behavior.*

Perfectionist personality refers to a trait characterized by the pursuit of excessively high standards (X. G. Zhang & Wang, 2018). Individuals with this trait typically impose extremely high expectations and criteria on themselves or others, struggling to tolerate any flaws or mistakes. Depending on the target of these demands, perfectionist personality can be further categorized into self-oriented perfectionism and other-oriented perfectionism (Stoeber, 2015).

Other-oriented perfectionism directs perfectionistic demands toward others rather than oneself (Hewitt & Flett, 1991). When leaders exhibit this trait, they tend to impose unattainably high standards on subordinates, closely monitoring and critically evaluating their performance. If subordinates fail to meet these expectations, the leader may display disappointment, blame, or even hostility (L. Guo et al., 2020). This perfectionist disposition leads leaders to be overly critical and demanding in the workplace, making subordinates feel oppressed, unappreciated, or incapable of meeting the leader’s expectations. Thus, a leader’s other-oriented perfectionism can also contribute to the emergence of exploitative leadership behavior. Accordingly, this study proposes the following hypothesis:

**H2:**  *A leader’s other-oriented perfectionism positively predicts their exploitative leadership behavior.*

Power distance orientation measures an individual’s acceptance of hierarchical power disparities or unequal power distribution within organizations (Dorfman & Howell, 1988; Hofstede, 1991). Leaders with a high-power distance orientation perceive authority and status differences across organizational levels as legitimate, thereby more readily emphasizing their own authority and status to secure subordinates’ compliance with their demands.

Consequently, such leaders tend to adopt authoritarian and dictatorial leadership styles (Koslowsky et al., 2011). They are reluctant to delegate authority, preferring to make decisions unilaterally and issue direct commands. Additionally, leaders with high power distance orientation pay minimal attention to employees’ emotional needs. They take subordinates’ hard work for granted, rarely providing material or psychological recognition, and show little concern for employees’ professional growth. When subordinates underperform, these leaders often express dissatisfaction through criticism, reduced support, heightened surveillance, or even withholding performance-based rewards. Thus, a leader’s high-power distance orientation can also drive exploitative behavior. Accordingly, this study proposes the following hypothesis:

**H3:**  *A leader’s high-power distance orientation positively predicts their exploitative leadership behavior.*

### 2.2. The Predictive Effect of Organizational Contextual Factors on Exploitative Leadership Behavior

In addition to leaders’ personality traits, organizational contextual factors may also contribute to the emergence of exploitative leadership behaviors. The following section examines how contextual factors such as task challenge, organizational status conferral mechanisms, and leader–subordinate goal misalignment predict exploitative leadership behavior.

Task challenge reflects the level of demands placed on organizational members’ capabilities and effort. When confronted with highly challenging tasks, leaders may experience heightened anxiety and stress, potentially leading them to adopt more extreme leadership approaches, including exploitative behaviors. Specifically, leaders might intensify control over and exploitation of employees to ensure task completion, often at the expense of employee well-being and long-term interests. This aligns with existing research demonstrating that leaders’ performance pressure (Deng et al., 2023) and perceptions of goal obstruction (Cohen & Baruch, 2022; Emmerling et al., 2023) can trigger exploitative leadership behaviors. Thus, we propose the following hypothesis:

**H4:**  *Task challenge positively predicts exploitative leadership behavior.*

Organizational status conferral ways refer to the core evaluation criteria and institutional mechanisms by which organizations allocate status symbols (e.g., promotions, salaries, authority, and organizational support). They reflect the relative emphasis placed on ascriptive factors (e.g., seniority, age, tenure, background) versus achieved factors (e.g., performance, competence, expertise, influence) in organizational design (Z. Q. Liu et al., 2015). Based on the dominant evaluation criteria, status conferral ways can be categorized into two types as follows:

Sticky status conferral: status is anchored in ascriptive factors. As a result, status is relatively fixed—once conferred, it becomes resistant to change.

Flexible status conferral: status is primarily tied to achieved factors. As a result, status is fluid—individuals can rapidly gain/lose standing based on current outputs.

Compared to sticky status conferral, flexible status conferral places greater emphasis on evaluating individual performance outputs. Consequently, under such systems, leaders may engage in exploitative behaviors—such as coercing overtime, exerting excessive pressure, or claiming undue credit—to achieve visible accomplishments and secure career advancement. Thus, we propose the following hypothesis:

**H5:**  *Flexible status conferral positively predicts exploitative leadership behavior.*

Leader–subordinate goal misalignment refers to the phenomenon where discrepancies or inconsistencies exist between leaders and subordinates regarding goal setting, interpretation, or implementation within an organization or team (Li et al., 2019). When such misalignment occurs, leaders may resort to pressuring, threatening, or intimidating subordinates to drive goal attainment due to ineffective communication and coordination (Padilla et al., 2007). This coercive approach neglects subordinates’ intrinsic needs and motivations, focusing solely on extrinsic incentives and outcomes, thereby constituting exploitative behavior. Accordingly, we propose the following hypothesis:

**H6:**  *Leader–subordinate goal misalignment positively predicts exploitative leadership behavior.*

### 2.3. The Activating Effect of Organizational Context on Leaders’ Personality Traits

Trait activation theory posits that personality traits manifest in individuals’ work behaviors through trait activation, which occurs in response to trait-relevant situational cues. These cues originate from three levels: task, social, and organizational (Tett & Burnett, 2003). This paper proposes that task challenge, leader–subordinate goal misalignment, and flexible status conferral represent situational cues at the task, social, and organizational levels, respectively. These cues activate leaders’ personality traits—namely self-interest, other-oriented perfectionism, and high-power distance orientation—thereby driving them to engage in exploitative leadership behaviors. The following section elaborates on this mechanism.

#### 2.3.1. The Moderating Role of Task Challenge in Trait Activation

As a task-level situational cue, task challenge serves as a critical factor in activating leaders’ personality traits and triggering exploitative leadership behaviors. When confronted with highly challenging tasks, self-interested leaders may intensify control over and exploitation of subordinates, coercing higher work output to ensure task completion, thereby alleviating their own anxiety and stress (Alajhar et al., 2024). Other-oriented perfectionist leaders tend to escalate expectations toward team members. Unmet expectations often provoke disappointment, anger, or frustration, manifesting as exploitative behaviors like excessive criticism, undue pressure, or neglect of team members’ needs (Hewitt & Flett, 1991). High power distance-oriented leaders become more likely to reinforce hierarchical authority. They emphasize power disparities through domineering behaviors such as micromanagement, restricted communication, dismissal of feedback, and denial of subordinate autonomy. These patterns demonstrate how task challenge activates three distinct personality traits (self-interest, other-oriented perfectionism, and high-power distance orientation), collectively predisposing leaders toward exploitative conduct. We therefore hypothesize:

**H7:**  *Task challenge positively moderates the relationships between three personality traits (self-interested personality/other-oriented perfectionism/high power distance orientation) and exploitative leadership behavior.*

#### 2.3.2. The Moderating Role of Leader–Subordinate Goal Misalignment in Trait Activation

As a social-level situational cue, leader–subordinate goal misalignment similarly activates leaders’ personality traits, predisposing them to exploitative leadership behaviors. When goal misalignment occurs, self-interested and high-power distance-oriented leaders may perceive their authority as being challenged. To safeguard their power interests, they typically enforce unilateral compliance through coercive measures, and suppress dissent by overtly asserting hierarchical dominance (Padilla et al., 2007). These actions create subordinates’ pervasive sense of exploitation.

Leaders with other-oriented perfectionism typically interpret goal misalignment as subordinates’ failure to meet expectations, triggering intense disappointment and anger. This emotional response often manifests through hypercritical evaluations of subordinate performance, excessive performance pressure tactics, systematic neglect of subordinate welfare (L. Guo et al., 2020). They may also forcibly realign subordinates’ efforts with their own standards to compensate for perceived losses.

In summary, goal misalignment activates and intensifies three personality traits—self-interest, other-oriented perfectionism, and high-power distance orientation—thereby exacerbating their propensity for exploitative leadership. Accordingly, we hypothesize:

**H8:**  *Leader–subordinate goal misalignment positively moderates the relationships between three personality traits (self-interested personality/other-oriented perfectionism/high power distance orientation) and exploitative leadership behavior.*

#### 2.3.3. The Moderating Role of Flexible Status Conferral in Trait Activation

As an organizational-level situational cue, flexible status conferral similarly activates leaders’ personality traits and facilitates the emergence of exploitative leadership behaviors. When organizational status allocation emphasizes “performance” over “seniority”, it typically induces status anxiety and pressure among leaders due to position instability (Z. Q. Liu et al., 2015). To reduce these effects, self-interested and high-power distance-oriented leaders may intensify control over and exploitation of subordinates to enhance performance output, viewing subordinates as tools to achieve personal goals while disregarding their rights and needs.

Leaders with other-oriented perfectionism tend to have higher expectations of their subordinates. They rely on their subordinates to maintain their position and push them to meet their expectations through excessive demands, criticism, and supervision (Hewitt & Flett, 1991). They will also view the failures and shortcomings of their subordinates as obstacles to achieving their goals, and continuously evaluate their performance negatively (L. Guo et al., 2020).

In summary, flexible status conferral activates and amplifies three key personality traits—self-interest, other-oriented perfectionism, and high-power distance orientation—thereby increasing the likelihood of exploitative leadership behaviors. Accordingly, we hypothesize:

**H9:**  *Flexible status conferral positively moderates the relationship between three personality traits (self-interested personality/other-oriented perfectionism/high power distance orientation) and exploitative leadership behavior.*

**Figure 2:**
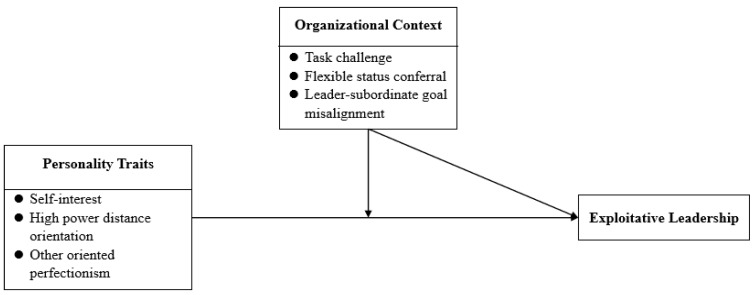
Conceptual model diagram of the causes and mechanisms of exploitative leadership.

Integrating the above propositions, Figure 2 presents the conceptual model illustrating the formative mechanisms underlying exploitative leadership.

## 3. Materials and Methods

### 3.1. Participants and Procedures

This study employed a two-phase online questionnaire approach to collect research data in China between November and December 2024. The survey was administered primarily on the Chinese professional online survey platform Wenjuanxing (https://www.wjx.cn/), which has a membership base of 6.2 million, with a nearly balanced gender distribution between male and female users. The platform’s members come from all regions of China and engage in various occupations, and over 90% of Chinese universities are currently using its data services. The leader–subordinate dyadic samples were recruited through paid services, with all participants required to complete real-name registration and pass identity verification for matching purposes.

The questionnaire employed a leader–subordinate dyadic design. In the first phase (T1), leaders were asked to report demographic information and complete scales measuring self-interested personality, high power distance orientation, other-oriented perfectionism, task challenge, flexible status conferral, and leader–subordinate goal misalignment. Two weeks later, in the second phase (T2), subordinates reported their demographic information and completed the exploitative leadership scale.

During T1, survey invitations were sent to 600 potential respondents (leaders/supervisors), with 518 questionnaires returned. After excluding 23 invalid responses based on screening questions, 495 valid questionnaires were retained. In T2, questionnaires were distributed to the 495 subordinates matched with the valid leader responses, yielding 458 returned questionnaires. After further excluding 36 invalid responses through screening procedures, a final sample of 422 valid matched dyads was obtained. The demographic characteristics of the final sample are presented in Table 1.

As shown in Table 1, in the leader sample, males accounted for 55.2%, with the majority aged between 36 and 45 (57.1%). The educational attainment was predominantly bachelor’s degree (38.6%), and organizational tenure was concentrated in 5–7 years (35.3%) and 7–10 years (36.3%). In the subordinate sample, males constituted 47.9%, with the majority aged between 26 and 35 (57.6%). Educational attainment was also primarily bachelor’s degree (40.5%), and organizational tenure was mainly 3–5 years (33.9%) and 5–7 years (36.0%). Additionally, in terms of industry distribution, the information transmission, software, and technology services sector accounted for 3.6%, education for 8.8%, finance for 10.0%, real estate for 15.6%, manufacturing for 41.0%, transportation, storage, and postal services for 13.7%, and other industries for 7.3%.

### 3.2. Measures

This study employed well-established scales from existing literature as measurement tools. To ensure measurement accuracy, two management Ph.D. holders conducted rigorous translation and back-translation of the scales to maintain both precision and readability. Unless otherwise specified, all scales used in this study adopted a 5-point Likert scale design, where 1 indicates “strongly disagree” and 5 indicates “strongly agree”.

Self-interested Personality (T1). Adapted from the self-interest preference scale developed by De Dreu and Nauta (2009), this construct was measured using 3 items. A sample item is: “At work, I am concerned about my own needs and interests.” In this study, the scale’s Cronbach’s α was 0.93.

High Power Distance Orientation (T1). This construct was measured using the 6-item scale proposed by Dorfman and Howell (1988). A sample item is: “I don’t think managers need to consult their subordinates when making decisions.” The scale demonstrated a Cronbach’s α of 0.94 in this study.

Other-oriented Perfectionism (T1). Assessed with the 5-item scale developed by Hewitt and Flett (1991). A sample item is: “I have high expectations for the people who are important to me.” The scale’s Cronbach’s α was 0.88 in this study.

Task Challenge (T1). Measured using the scale developed by Withey et al. (1983), with the three items exhibiting the highest factor loadings selected for assessment. A sample item is: “Only a few tasks involve routine work with fixed operating procedures.” The scale’s Cronbach’s α was 0.92 in this study.

Flexible Status Conferral (T1). Assessed with the 4-item scale developed by Z. Q. Liu et al. (2015). A sample item (reverse-coded) is: “In our organization, promotion is based on seniority rather than meritocracy.” The scale’s Cronbach’s α was 0.89.

Leader–Subordinate Goal Misalignment (T1). Adapted from Uhl-Bien et al. (2022), this construct was measured using 3 contextually relevant items. A sample item (reverse-coded) is: “Department members’ personal goals are consistent with our department’s goals.” The scale’s Cronbach’s α was 0.89.

Exploitative Leadership (T2). Measured using Schmid et al.’s (2019) 15-item scale, which includes five dimensions: genuine egoistic behaviors, exerting pressure, undermining development, taking credit, and manipulative leader behavior. A sample item is: “My leader takes it for granted that my work can be used for his/her personal benefit.” The scale demonstrated a Cronbach’s α of 0.89 in this study.

Additionally, drawing on prior relevant research, this study incorporates demographic variables—including leader gender, age, education level, work tenure, organizational tenure, and industry type—as control variables (T1). The complete versions of the scales used in this study are provided in the Appendix A.

## 4. Results

### 4.1. Common Method Bias Test

To address potential common method bias, Harman’s single-factor test was conducted. The results showed that the first unrotated principal component accounted for 27.20% of the variance, which did not exceed the 40% threshold. Additionally, confirmatory factor analysis (CFA) indicated poor fit for the single-factor model (*χ*^2^/*df* = 11.77, *CFI* = 0.39, *TLI* = 0.35, *IFI* = 0.39, *RMSEA* = 0.16, *RMR* = 0.18). 

Subsequently, a common method factor was introduced for further examination. The results demonstrated significantly improved model fit indices (*χ*^2^/*df* = 1.69, *CFI* = 0.97, *TLI* = 0.96, *IFI* = 0.97, *RMSEA* = 0.04, *RMR* = 0.04). However, when compared with the theoretical model, the changes in key indices were all less than 0.02 (Δ*CFI* = 0.01, Δ*TLI* = 0.01, Δ*IFI* = 0.01, Δ*RMSEA* = −0.004, Δ*RMR* = −0.007). These results collectively indicate that common method bias is not a substantial concern in the current study.

### 4.2. Confirmatory Factor Analysis

This study employed confirmatory factor analysis (CFA) to examine the discriminant validity of the variables. Given the multidimensional nature and excessive number of items in the exploitative leadership scale, we followed established research practices by first conducting item parceling for this construct. The CFA results (Table 2) demonstrated that only the seven-factor model met acceptable statistical thresholds (*χ*^2^/*df* = 2.20, *CFI* = 0.96, *TLI* = 0.95, *IFI* = 0.96, *RMSEA* = 0.05), indicating adequate discriminant validity among the core variables and supporting further analysis.

### 4.3. Descriptive Statistics

The means, standard deviations, and correlation coefficients of all variables are presented in Table 3. As shown in the table, the correlation coefficients between each variable and others were all smaller than the square roots of their average variance extracted ($\sqrt{AVE}$), providing further evidence for satisfactory discriminant validity among the core variables in this study. Additionally, exploitative leadership showed significant positive correlations with self-interested personality (*r* = 0.15, *p* < 0.01), other-oriented perfectionism (*r* = 0.27, *p* < 0.001), high-power distance orientation (*r* = 0.29, *p* < 0.001), task challenge (*r* = 0.19, *p* < 0.001), flexible status conferral (*r* = 0.14, *p* < 0.01), and leader–subordinate goal misalignment (*r* = 0.23, *p* < 0.001). These results provide preliminary support for Hypotheses 1 to 6 of this study.

### 4.4. Hypothesis Testing

To test the research hypotheses, regression analysis was employed. After controlling for leader gender, age, education level, work tenure, organizational tenure, and industry type, the regression results are presented in Table 4.

As shown in Table 4, regarding direct effects, self-interested personality demonstrated a significant positive influence on exploitative leadership (*β* = 0.16, *p* < 0.01), supporting Hypothesis 1. Other-oriented perfectionism showed a significant positive effect on exploitative leadership (*β* = 0.26, *p* < 0.001), supporting Hypothesis 2. High-power distance orientation exhibited a significant positive impact on exploitative leadership (*β* = 0.29, *p* < 0.001), supporting Hypothesis 3. Task challenge had a significant positive influence on exploitative leadership (*β* = 0.18, *p* < 0.001), supporting Hypothesis 4. Flexible status conferral demonstrated a significant positive effect on exploitative leadership (*β* = 0.13, *p* < 0.01), supporting Hypothesis 5. Leader–subordinate goal misalignment showed a significant positive effect on exploitative leadership (*β* = 0.24, *p* < 0.001), supporting Hypothesis 6.

**Figure 3:**
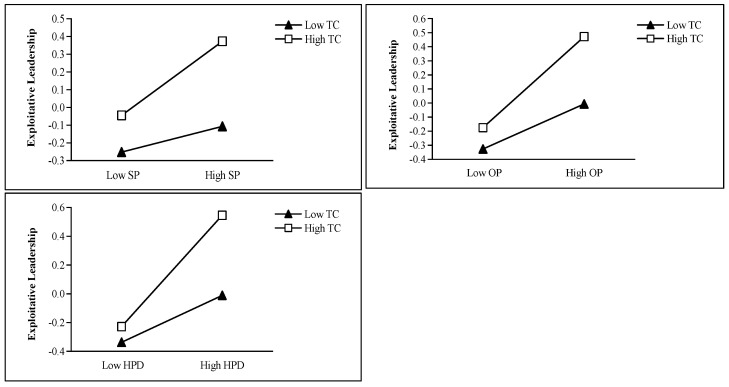
Simple slope plots of the moderating effects of task challenge. Note. SP means self-interested personality, OP means other-oriented perfectionism, HPD means high-power distance orientation, TC means task challenge.

Regarding moderating effects, task challenge showed a significant positive moderating effect on the relationship between high-power distance orientation and exploitative leadership (*β* = 0.11, *p* < 0.05). However, it failed to reach statistical significance in moderating the relationships between self-interested personality and exploitative leadership (*β* = 0.07, *p* = 0.172) or between other-oriented perfectionism and exploitative leadership (*β* = 0.08, *p* = 0.078), indicating that Hypothesis 7 received only partial support. The simple slope plots of the moderating effects of task challenge are shown in Figure 3.

**Figure 4:**
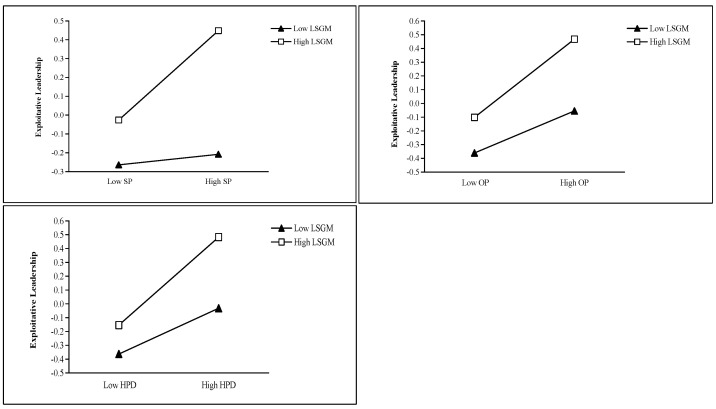
Simple slope plots of the moderating effects of leader–subordinate goal misalignment. Note. SP means self-interested personality, OP means other-oriented perfectionism, HPD means high-power distance orientation, LSGM means leader–subordinate goal misalignment.

Leader–subordinate goal misalignment demonstrated a significant positive moderating effect on the relationship between self-interested personality and exploitative leadership (*β* = 0.10, *p* < 0.05). Nevertheless, it failed to show significant moderating effects on the relationships between other-oriented perfectionism and exploitative leadership (*β* = 0.07, *p* = 0.142) or between high-power distance orientation and exploitative leadership (*β* = 0.08, *p* = 0.089), suggesting that Hypothesis 8 likewise obtained only partial support. The simple slope plots of the moderating effects of leader–subordinate goal misalignment are shown in Figure 4.

**Figure 5:**
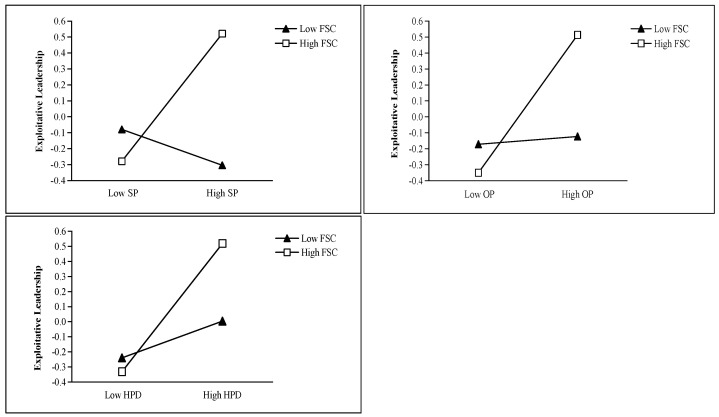
Simple slope plots of the moderating effects of flexible status conferral. Note. SP means self-interested personality, OP means other-oriented perfectionism, HPD means high-power distance orientation, FSC means flexible status conferral.

Flexible status conferral exhibited significant positive moderating effects on the relationships of exploitative leadership with self-interested personality (*β* = 0.26, *p* < 0.001), other-oriented perfectionism (*β* = 0.20, *p* < 0.001), and high-power distance orientation (*β* = 0.15, *p* < 0.001), providing full support for Hypothesis 9. The simple slope plots of the moderating effects of flexible status conferral are shown in Figure 5.

The results of our hypothesis testing are summarized in Table 5. As shown in the table, while most hypotheses were supported, some moderating effect hypotheses failed to achieve statistical significance. Below we provide further analysis of these unsupported hypotheses.

First, regarding the non-significant moderating effects of task challenge on the relationships between self-interested personality/other-oriented perfectionism and exploitative leadership (Hypothesis 7), we propose that these personality traits may inherently contain stable tendencies toward exploitation and excessive demands. Task challenge might primarily alter the manifestation rather than the intensity of exploitation. For instance, as task challenge increases, leaders with self-interested personality may shift their exploitative behaviors from seeking additional benefits (e.g., appropriating subordinates’ achievements) to risk transfer (e.g., making subordinates accountable for failures). Leaders with other-oriented perfectionism may transition their focus from detail perfection (e.g., criticizing report formatting errors) to outcome perfection (e.g., insisting on exceeding targets). In both cases, the fundamental degree of exploitation remains relatively unchanged, merely expressed through different behavioral patterns.

Second, concerning the non-significant moderating effect of leader–subordinate goal misalignment on the relationship between other-oriented perfectionism and exploitative leadership (Hypothesis 8), we similarly argue that goal misalignment may only modify the specific manifestations of exploitative behaviors among perfectionist leaders rather than altering their intensity. For instance, such leaders might shift from criticizing subordinates’ work efficiency to reproaching their work attitudes, while maintaining equivalent levels of exploitation. Regarding the non-significant moderating effect of goal misalignment on the relationship between high-power distance orientation and exploitative leadership, this study posits that the exploitative behaviors of high-power distance-oriented leaders stem from their absolute belief in hierarchical authority, which likely exhibits low situational sensitivity. For instance, when goals are aligned, high power distance-oriented leaders may employ coercive measures to maintain order (e.g., “You must do it my way”); when goals are misaligned, they may use coercion to suppress dissent (e.g., “How dare you challenge my objectives?”). This reveals that coercion and pressure are their default means of managing subordinates, with goal misalignment merely altering the reasons for exploiting subordinates rather than the intensity of such exploitation.

## 5. Discussion

Guided by the interactionist perspective and trait activation theory, this study examines the antecedents and formation mechanisms of exploitative leadership through the dual lenses of leader personality traits and organizational contexts. The findings reveal that: leader personality traits—including self-interested personality, other-oriented perfectionism, and high-power distance orientation—have significant positive effects on exploitative leadership behaviors. Organizational contexts—such as task challenge, flexible status conferral, and leader–subordinate goal misalignment—also have significant positive influences on exploitative leadership behaviors. Flexible status conferral significantly strengthens the positive relationships between all three personality traits and exploitative leadership; task challenge and leader–subordinate goal misalignment, respectively, amplify the positive relationships of exploitative leadership with high power distance orientation and self-interested personality.

### 5.1. Theoretical Contributions

Distinct from prior studies focusing on the consequences of exploitative leadership, this study responds to recent calls in the literature (L. Y. Guo et al., 2023) by investigating its antecedents and formation mechanisms. The theoretical contributions of this study are reflected in three aspects:

First, this research enriches extant exploitative leadership theory by identifying its key triggers. As noted earlier, while existing studies have predominantly examined the outcomes of exploitative leadership and made some progress in understanding its causes, significant gaps remain. By empirically validating three leader personality traits (self-interested personality, other-oriented perfectionism, and high-power distance orientation) and three organizational contexts (task challenge, flexible status conferral, and leader–subordinate goal misalignment) as significant predictors of exploitative leadership, this study substantially addresses current theoretical deficiencies regarding its antecedents, representing an important advancement in exploitative leadership research.

Second, the study extends exploitative leadership theory by elucidating its formation mechanisms. Our findings demonstrate that organizational contexts activate leader personality traits in the development of exploitative leadership. For instance, when organizational status conferral emphasizes “performance” over “seniority” (i.e., flexible status conferral), leaders with self-interested personalities, other-oriented perfectionism, or high-power distance orientation are more likely to exhibit exploitative behaviors, as the context further activates these inherent traits. This discovery not only deepens scholarly understanding of how exploitative leadership emerges but also provides robust validation for trait activation theory.

Third, diverging from the conventional view that “situational cues activate personality traits, thereby increasing the frequency or intensity of related behaviors” (e.g., Tett & Guterman, 2000), this study proposes that “the consequences of trait activation may manifest as either increased behavioral intensity or transformations in behavioral forms”, thereby providing a valuable extension to trait activation theory. Consistent with this view, self-interested individuals in high-monitoring environments may shift from outright embezzlement to exploiting institutional loopholes rather than reducing corrupt behaviors. Perfectionists unable to criticize subordinates (e.g., due to organizational prohibitions) may redirect their tendencies toward excessive procedural monitoring. Machiavellian traits activated in low-justice contexts may result in covert manipulation (e.g., information control) replacing overt exploitation (public coercion). This aligns with the adaptive principle from evolutionary psychology: shifts in behavioral forms constitute survival strategies for responding to environmental pressures (Buss, 2009).

### 5.2. Practical Implications

This study yields the following practical implications:

First, regarding leader personality traits, our findings demonstrate that self-interested personality, other-oriented perfectionism, and high-power distance orientation all significantly and positively predict exploitative leadership. Therefore, organizations should: (1) incorporate personality assessment (e.g., using the measurement scales adopted in the current study) in leader selection and promotion processes to identify and avoid candidates with these predispositions; and (2) provide targeted training programs to mitigate these traits among existing leaders, thereby reducing the occurrence of exploitative leadership behaviors.

Second, regarding organizational contexts, factors such as task challenge, flexible status conferral, and leader–subordinate goal misalignment not only directly led to exploitative leadership but also amplify the influence of the aforementioned personality traits. Accordingly, we recommend the following management practices: (1) rationally regulating task difficulty to alleviate excessive performance pressure on leaders; (2) refining status allocation mechanisms by incorporating employee satisfaction metrics into evaluation systems; and (3) enhancing leader–subordinate communication to align objectives and expectations.

### 5.3. Limitations and Future Research

While this study has identified several antecedents of exploitative leadership, it has merely revealed the “tip of the iceberg” regarding its formation mechanisms. Compared with the substantial body of research on its consequences, scholarly understanding of its causes and developmental processes remains limited and requires further refinement. Future studies should therefore conduct more in-depth investigations into the antecedents of exploitative leadership.

Moreover, the current conceptualization and measurement scales of exploitative leadership employed in academia were developed by German scholars Schmid et al. (2019) within their national context. However, given the substantial differences in cultural, institutional, and ideological dimensions across countries (e.g., between Germany and China), the universal applicability of the conceptualization and measurement scales to other national or cultural contexts remains to be empirically verified. 

For instance, compared with Germany (Hofstede: Power Distance = 35; Individualism = 67), China’s higher power distance (Hofstede: Power Distance = 80) and collectivistic culture (Hofstede: Individualism = 20) may attenuate employees’ perception of leaders’ exploitative behaviors (e.g., leaders taking credit for subordinates’ work). In other words, within Chinese organizational contexts, certain dimensions of existing constructs may not necessarily be perceived as exploitative by subordinates. Consequently, future research should focus on developing indigenous conceptualizations, measurement scales, and investigating antecedent-consequence relationships of exploitative leadership, with the ultimate aim of establishing a theoretical framework that is properly contextualized to the organizational environments of respective nations.

## 6. Conclusions

Based on trait activation theory, this study constructs a moderated effect model to uncover the causes and formation mechanisms of exploitative leadership. Based on empirical tests of questionnaire data from 422 leader–subordinate dyads, the research results basically support the predictive effects of three leader personality traits (i.e., self-interested personality, others-oriented perfectionism, and high-power distance orientation) and three contextual factors (i.e., task challenge, flexible status conferral, and leader–subordinate goal misalignment) on exploitative leadership behavior, along with their potential trait-activating effects. Building upon these results, we discuss the theoretical contributions and practical implications of our research, while also suggesting promising avenues for future investigations.

## Figures and Tables

**Table 1:** Sample structure.

Leader Sample Structure			Subordinate Sample Structure		
Variable	Category	Percentage	Variable	Category	Percentage
Gender	Male	55.2%	Gender	Male	47.9%
	Female	44.8%		Female	52.1%
Age	≤25 years old	5.2%	Age	≤25 years old	10.0%
	26–35 years old	32.7%		26–35 years old	57.6%
	36–45 years old	57.1%		36–45 years old	31.0%
	≥46 years old	5.0%		≥46 years old	1.4%
Education	Master’s degree	3.3%	Education	Master’s degree	26.6%
	Bachelor’s degree	38.6%		Bachelor’s degree	40.5%
	Associate degree	35.8%		Associate degree	32.2%
	High school and below	22.3%		High school and below	0.7%
Organizational Tenure	≤3 years	3.8%	Organizational Tenure	≤3 years	15.7%
	3–5 years	6.4%		3–5 years	33.9%
	5–7 years	35.3%		5–7 years	36.0%
	7–10 years	36.3%		7–10 years	11.1%
	≥10 years	18.2%		≥10 years	3.3%

**Table 2:** Confirmatory factor analysis.

Model	*χ* ^2^	*df*	*χ*^2^/*df*	*CFI*	*TLI*	*IFI*	*RMSEA*
Seven-factor model (*E*, *S*, *O*, *H*, *T*, *F*, *L*)	725.11	329	2.20	0.96	0.95	0.96	0.05
Six-factor model (*E*, *S*, *O + H*, *T*, *F*, *L*)	1042.99	335	3.11	0.92	0.91	0.92	0.07
Six-factor model (*E+S*, *O*, *H*, *T*, *F*, *L*)	1224.12	335	3.65	0.90	0.89	0.90	0.08
Six-factor model (*E*, *O*, *S + H*, *T*, *F*, *L*)	1411.69	335	4.21	0.88	0.87	0.88	0.09
Five-factor model (*E + O*, *S + H*, *T*, *F*, *L*)	1541.89	340	4.54	0.87	0.85	0.87	0.09
Five-factor model (*E*, *S + O + H*, *T, F*, *L*)	1673.64	340	4.92	0.85	0.84	0.85	0.10
Four-factor model (*E*, *S + O + H*, *T + F*, *L*)	1983.95	344	5.77	0.82	0.80	0.82	0.11
Three-factor model (*E*, *S + O + H*, *T + F + L*)	2518.82	347	7.26	0.76	0.74	0.76	0.12
Two-factor model (*E*, *S + O + H + T + F + L*)	4599.49	349	13.18	0.53	0.49	0.53	0.17
One-factor model (*E + S + O + H + T + F + L*)	5004.08	350	14.30	0.48	0.44	0.48	0.18

Note: *E* stands for exploitative leadership, *S* stands for self-interested personality, *O* stands for other-oriented perfectionism, *H* stands for high-power distance orientation, *T* stands for task challenge, *F* stands for flexible status conferral, and *L* stands for leader–subordinate goal misalignment.

**Table 3:** Descriptive statistics and correlation coefficient table.

	Mean	SD	1	2	3	4	5	6	7
Exploitative leadership	3.16	0.59	**0.68**						
Self-interested personality	3.04	1.30	0.15 **	**0.91**					
Other-oriented perfectionism	2.53	1.16	0.27 ***	0.64 ***	**0.78**				
High-power distance orientation	2.75	1.28	0.29 ***	0.65 ***	0.76 ***	**0.86**			
Task challenge	3.74	0.86	0.19 ***	0.12 *	0.11 *	0.07	**0.89**		
Flexible status conferral	3.56	0.95	0.14 **	0.14 **	0.16 **	0.08	0.70 ***	**0.87**	
Leader–subordinate goal misalignment	3.19	0.89	0.23 ***	0.12 *	0.18 ***	0.22 ***	0.51 ***	0.51 ***	**0.86**

Note: * indicates *p* < 0.05, ** indicates *p* < 0.01, and *** indicates *p* < 0.001. The bold values on the diagonal represent $\sqrt{AVE}$.

**Table 4:** Regression analysis results.

	Exploitative Leadership			
	*β*	*S.E.*	*p*	*95% CI*
**Direct effect**				
Self-interested personality	0.16	0.05	0.001	[0.06, 0.25]
Other-oriented perfectionism	0.26	0.05	0.000	[0.17, 0.35]
High-power distance orientation	0.29	0.05	0.000	[0.19, 0.38]
Task challenge	0.18	0.05	0.000	[0.08, 0.27]
Flexible status conferral	0.13	0.05	0.008	[0.03, 0.22]
Leader–subordinate goal misalignment	0.24	0.05	0.000	[0.15, 0.33]
**Moderating effect**				
Self-interested personality × Task challenge	0.07	0.05	0.172	[−0.03, 0.17]
Other-oriented perfectionism × Task challenge	0.08	0.05	0.078	[−0.01, 0.17]
High-power distance orientation × Task challenge	0.11	0.05	0.016	[0.02, 0.20]
Self-interested personality × Flexible status conferral	0.26	0.05	0.000	[0.16, 0.35]
Other-oriented perfectionism × Flexible status conferral	0.20	0.04	0.000	[0.12, 0.29]
High-power distance orientation × Flexible status conferral	0.15	0.05	0.000	[0.06, 0.24]
Self-interested personality × Leader–subordinate goal misalignment	0.10	0.05	0.029	[0.01, 0.20]
Other-oriented perfectionism × Leader–subordinate goal misalignment	0.07	0.04	0.142	[−0.02, 0.15]
High-power distance orientation × Leader–subordinate goal misalignment	0.08	0.04	0.089	[−0.01, 0.16]

**Table 5:** Summary table of hypothesis test results.

Research Hypothesis	Results
**H1.** *A leaders’ self-interested personality positively predicts their exploitative leadership behavior.*	Supported
**H2.** *A leader’s other-oriented perfectionism positively predicts their exploitative leadership behavior.*	Supported
**H3.** *A leader’s high-power distance orientation positively predicts their exploitative leadership behavior.*	Supported
**H4.** *Task challenge positively predicts exploitative leadership behavior.*	Supported
**H5.** *Flexible status conferral positively predicts exploitative leadership behavior.*	Supported
**H6.** *Leader–subordinate goal misalignment positively predicts exploitative leadership behavior.*	Supported
**H7.** *Task challenge positively moderates the relationships between three personality traits (self-interested personality/other-oriented perfectionism/high power distance orientation) and exploitative leadership behavior.*	Partially supported
**H8.** *Leader–subordinate goal misalignment positively moderates the relationships between three personality traits (self-interested personality/other-oriented perfectionism/high power distance orientation) and exploitative leadership behavior.*	Partially supported
**H9.** *Flexible status conferral positively moderates the relationship between three personality traits (self-interested personality/other-oriented perfectionism/high power distance orientation) and exploitative leadership behavior.*	Supported

## Data Availability

The data that support the findings of this study are available from the corresponding author, Yilin Zhang, upon reasonable request.

## Appendix A

The detailed measurement scales used in the current study are as follows.

**Table A1:** Core variables and their measurement items.

Variable	Measurement Items
Self-interested Personality	At work, I am concerned about my own needs and interests.
	At work, my personal goals and aspirations are important to me.
	At work, I consider my own wishes and desires to be relevant.
High-power Distance Orientation	I don’t think managers need to consult their subordinates when making decisions.
	I think it is often necessary for managers to exercise power and exert authority at work.
	I think managers should not exchange opinions excessively with employees.
	I think supervisors should avoid non-work-related interactions with subordinates.
	I think subordinates should not question or oppose the decisions made by superiors.
	I think superiors should not delegate important tasks to subordinates for resolution.
Other-oriented Perfectionism	I have high expectations for the people who are important to me.
	I do not have very high standards for those around me. (Reverse)
	If I ask someone to do something, I expect it to be done flawlessly.
	I can’t be bothered with people who won’t strive to better themselves.
	The people who matter to me should never let me down.
Task challenge	Only a few jobs are the same from day-to-day.
	Only a few tasks involve routine work with fixed operating procedures.
	Department members rarely use identical approaches to complete the same tasks.
Flexible Status Conferral	In our organization, promotion is based on seniority rather than meritocracy (Reverse).
	In our organization, newcomers are more likely to be dismissed (Reverse).
	In our organization, rewards are allocated by tenure rather than performance (Reverse).
	In our organization, ascribed factors primarily determine status attainment (Reverse).
Leader–Subordinate Goal Misalignment	Department members have the best interests of the department in mind (Reverse).
	Department members’ personal goals are consistent with our department’s goals (Reverse).
	Department members look out for our department’s best interests (Reverse).
Exploitative Leadership	My leader takes it for granted that my work can be used for his/her personal benefit. (genuine egoistic behaviors).
	My leader sees employees as a means to reach his/her personal goals. (genuine egoistic behaviors).
	My leader values the achievement of his/her own goals over the needs of the employees. (genuine egoistic behaviors).
	My leader puts me under pressure to reach his/her goals. (exerting pressure).
	My leader increases my workload without considering my needs in order to reach his/her goals. (exerting pressure).
	My leader does not consider my workload when new tasks need to be assigned. (exerting pressure).
	My leader gives me tedious tasks if he/she can benefit from it. (underchallenging followers).
	My leader does not give me opportunities to further develop myself professionally because his/her own goals have priority. (underchallenging followers).
	My leader gives me boring routine tasks when he/she can benefit from it. (underchallenging followers).
	My leader uses my work to get himself/herself noticed. (taking credit).
	My leader passes the team’s work off as his/her own. (taking credit).
	My leader uses my work for his/her personal gain. (taking credit).
	My leader plays my colleagues and me off against each other to reach his/her goals. (manipulating followers).
	My leader manipulates others to reach his/her goals. (manipulating followers).
	My leader does not hesitate to manipulate or deceive employees in order to reach his/her goals (manipulating followers).
